# Unravelling the Ties: How Attachment Styles and Emotion Regulation Fuel Emotional Eating in Youth With Obesity—A Clinical Sample Study

**DOI:** 10.1111/ijpo.70095

**Published:** 2026-02-09

**Authors:** Joana Gómez‐Odriozola, Jolien Braet, Ine Verbiest, Caroline Braet

**Affiliations:** ^1^ Department of Clinical and Health Psychology & Research Methods University of the Basque Country UPV/EHU Donostia‐San Sebastián Spain; ^2^ Department of Developmental, Personality, and Social Psychology Ghent University Ghent Belgium

**Keywords:** attachment, emotion regulation, emotional eating, obesity, youth

## Abstract

Emotional eating is critical in the development and maintenance of obesity among children and adolescents. While attachment's influence on emotional eating is increasingly recognised, little is known about how emotion regulation strategies mediate this, particularly in samples with obesity. This study examined how attachment dimensions affect emotional eating through different emotion regulation strategies in youths with obesity. 772 children and adolescents (ages 7–19) with obesity participated. Key variables were measured using validated questionnaires. Mediation effects were analysed through Structural Equation Modelling, with exploratory analyses assessing the role of the emotion regulation strategies diversity index and specific emotion regulation strategies. Higher attachment anxiety and avoidance were associated with greater emotional eating, both directly and indirectly through maladaptive emotion regulation strategies. Adaptive strategies did not show mediating effects. Attachment anxiety and avoidance increased the diversity of emotion regulation strategies, which was associated with higher emotional eating. Interventions may benefit from prioritising the effectiveness of these strategies and addressing maladaptive ones. Excessive diversity of emotion regulation strategies could reflect underlying difficulties and may be associated with higher emotional eating. A deeper understanding of the interplay between attachment and emotion regulation could inform more targeted approaches for preventing and treating obesity in youth.

## 1 Introduction

Emotional eating, defined as eating in response to emotional rather than physiological cues [1], is a clinically relevant behaviour in childhood and adolescence. Particularly among youths with elevated weight, this maladaptive eating behaviour is important, given its association with weight difficulties and long‐term health risks [2, 3]. Developmental research suggests that emotional eating often emerges or intensifies during adolescence [4]; however, the psychological mechanisms driving this behaviour in youths with obesity remain poorly understood.

The etiology of emotional eating reflects a complex interplay of psychological factors. From an attachment perspective, emotional eating may reflect a response to distress when interpersonal support is perceived as unavailable [5, 6]. Insecure attachment, encompassing attachment anxiety (fear of rejection, need for closeness) and attachment avoidance (preference for self‐reliance), is consistently linked to maladaptive eating behaviours, including emotional eating [7, 8]. In children with obesity, attachment insecurity predicts poorer eating self‐regulation and higher body mass index (BMI) [9]; nevertheless, the mechanisms linking attachment and emotional eating in clinical paediatric samples remain insufficiently studied [10].

One possible mechanism linking attachment to emotional eating is emotion regulation. Emotion regulation plays a central role in eating behaviour and obesity [11], and its influence on disordered eating is well established [1, 2]. The emotional bond between children and their caregivers shapes expectations of support and guides the development of emotion regulation processes [12]. Food may become conditioned as a means of soothing or escaping distressing emotions [13], and early affective experiences with caregivers can reinforce this pattern. For instance, Kaplan and Kaplan [14] proposed that emotional eating may develop when parents use food to comfort children, thereby associating feeding with love and comfort rather than nutritional need.

Within the broader emotion regulation literature, emotion regulation strategies are commonly conceptualised and operationalised as adaptive (e.g., problem solving) or maladaptive (e.g., rumination) [15]. Secure attachment fosters adaptive regulation, while insecure attachment is associated with reliance on maladaptive strategies, such as suppression, rumination, or emotional disengagement [6, 16, 17]. These patterns differ by attachment dimension: attachment anxiety is linked to heightened emotional reactivity and perseverative coping, while attachment avoidance is associated with emotional distancing and self‐reliance [6, 18]. These maladaptive regulatory patterns may increase the risk of emotional eating, particularly in youths with obesity. When effective emotion regulation skills are lacking, individuals may rely on palliative coping, using external regulators—such as substances, medication, or food—to attenuate emotional arousal rather than address its source [13]. Indeed, in youths, maladaptive strategies are associated with higher emotional eating whereas adaptive emotion regulation is associated with lower emotional eating [19, 20, 21, 22]. Additionally, prior research suggests that having access to a repertoire of diverse emotion regulation strategies can be greatly benefical [23, 24]. Emotion regulation diversity refers to the breadth and variability of an individual's regulatory repertoire, which may reflect either a rich and expansive set of strategies or, alternatively, a pattern of broad but unfocused strategy use that signals less effective regulation [24, 25].

Prior research suggests that attachment insecurity is associated with emotional eating through general difficulties in emotion regulation [16], as well as through specific emotion‐related processes, including emotional suppression and reduced emotional awareness (alexithymia) [26]. More recently, evidence indicates that rumination may play a role in the association between attachment anxiety and disordered eating under stress, whereas comparable pathways for attachment avoidance are less consistently supported [27]. Overall, this emerging evidence highlights that only a limited range of emotion regulation strategies has been examined to date in youths, that findings are largely based on community samples, and that attachment dimensions may be linked to emotional eating through partially distinct regulatory mechanisms.

The present study aimed to examine the association between insecure attachment and emotional eating in youths with obesity with a focus on the mediating role of emotion regulation strategies. Specifically, we investigated a range of representative adaptive (e.g., cognitive problem‐solving) and maladaptive (e.g., withdrawal) emotion regulation strategies. This study hypothesised that higher levels of attachment anxiety and avoidance would be associated with greater emotional eating, mediated by increased use of maladaptive and reduced use of adaptive emotion regulation strategies. Moreover, the study aimed to explore the relations between emotional eating, attachment, and the diversity in the use of specific emotion regulation strategies [24], thereby moving beyond traditional categorisations of these strategies as adaptive or maladaptive [15, 25].

## 2 Methods

### 2.1 Participants and Procedure

Participants were children and adolescents enrolled in an inpatient Multidisciplinary Obesity Treatment program between 2013 and 2018 and were recruited following referral to the treatment center. Eligibility criteria included an age between 7 and 19 years, a BMI above 30 kg/m^2^, and the presence of at least two obesity‐related physical comorbidities (e.g., sleep apnea). In addition, participants were Dutch‐ or French‐speaking youths with normal intelligence who were identified by the treatment program as primarily obese. Height and weight were measured during the first clinical assessment as part of the treatment program. Adjusted Body Mass Index (%BMI) was calculated as the ratio of measured BMI to the normative BMI for age and sex (50th percentile), multiplied by 100. This method accounts for differences in growth and development. In children, a %BMI score ≥ 120% is considered as overweight and a score ≥ 140% as obesity [28]. Adjusted BMI was calculated only for descriptive purposes and was not included in the mediation analyses, in line with the study's focus on psychological mechanisms and due to incomplete anthropometric data.

Psychosocial variables were assessed by an online questionnaire completed by the adolescents before treatment. This study conducted secondary analyses of data collected as described in Naets et al. [29], Verbiest et al. [30], and Vervoort et al. [31]. Ethical approval was obtained from the institutional research committee of the Ghent University Faculty of Psychology and Psychological Sciences (2015/88). The available sample size was sufficient to detect a mediation effect with a statistical power of 0.80, assuming small effect sizes for the associations between attachment and emotion regulation strategies and between emotion regulation strategies and emotional eating [32]. Participants provided written assent or consent based on their age, and one of their parents gave informed consent before data collection. The hypotheses and data analysis plan were pre‐registered on a public platform (https://doi.org/10.17605/OSF.IO/FKQCM).

### 2.2 Measures

#### 2.2.1 Emotional Eating

The Dutch Eating Behaviour Questionnaire (DEBQ [33]) was used to assess emotional eating. Youths rate items on a Likert scale (from 1 ‘*never*’ to 5 ‘*very often*’). Higher DEBQ scores represent more maladaptive eating. For this study, scores on the emotional eating subscale were used (13 items; e.g., ‘Do you have a desire to eat when you have nothing to do?’). For the present study, reliability was examined by computing McDonald's omega coefficients (based on recommendations from Hayes and Coutts [34]), yielding a score of 0.96 for emotional eating.

#### 2.2.2 Attachment

The Experiences in Close Relationships Scale–Revised Child version (ECR‐RC [35]) was used to assess two forms of insecure attachment to the mother: attachment anxiety (18 items; e.g., ‘I'm afraid my mother will stop loving me’) and avoidance (18 items; e.g., ‘I prefer not to get too close to my mother’). Youths rate items on a Likert scale (from 1 ‘*strongly disagree*’ to 7 ‘*strongly agree*’). The omega was 0.94 for both attachment anxiety and avoidance.

#### 2.2.3 Emotion Regulation Strategies

A Dutch version of the Fragebogen zur Erhebung der Emotionsregulation bei Kindern und Jugendlichen (FEEL‐KJ [36]) was used to assess emotion regulation strategies. It is a 90‐item questionnaire assessing the use of adaptive and maladaptive emotion regulation strategies in response to fear, sadness, and anger. Each item is rated on a Likert scale (from 1 ‘*almost never*’ to 5 ‘*almost always*’). The scale provides total scores representing general dispositions to adaptively or maladaptively cope with emotions. Specific adaptive subscales comprise behavioural problem‐solving (e.g., ‘I try to change what makes me angry’), cognitive problem‐solving (e.g., ‘I think about what I can do’), forgetting (e.g., ‘I think it will pass’), acceptance (e.g., ‘I accept what makes me angry’), distraction (e.g., ‘I do something fun’), humour enhancement (e.g., ‘I think about things that make me happy’), and reappraisal (e.g., ‘I tell myself it is nothing important’). Maladaptive subscales include giving up (e.g., ‘I don't want to do anything’), aggression (e.g., ‘I get into a quarrel with others’), perseveration (e.g., ‘I cannot get it out of my head’), self‐devaluation (e.g., ‘I blame myself’), and withdrawal (e.g., ‘I don't want to see anyone’). There are also three additional subscales: social support (e.g., ‘I tell someone how I am doing’), expression (e.g., ‘I express my anger’), and emotional control (e.g., ‘I keep my feelings for myself’). All the scales were used in this study. Omega coefficients yielded scores of 0.97 for adaptive strategies and 0.93 for maladaptive strategies, with coefficients ranging from 0.86 to 0.94 for each specific strategy.

##### 2.2.3.1 Diversity of Emotion Regulation Strategies

The diversity index, which assesses the frequency and relative abundance of emotion regulation strategies, was calculated using Wen et al. [24] approach, applying the Shannon entropy formula for Likert scales:

$$H=-\sum_{s=1}^{L}\;p_{s}\times \ln\left(p_{s}\right)$$

Each score for a strategy subscale was divided by the maximum possible total sum across the 15 emotion regulation strategies subscales (*p*_*s*_). The resulting proportion for each subscale was then multiplied by its natural logarithm. These products were summed for each strategy subscale and then multiplied by −1. Higher values represent greater diversity of emotion regulation strategies.

### 2.3 Data Analytic Plan

Prior to testing structural models, confirmatory factor analyses (CFA) were conducted for each questionnaire separately to validate their factor structure. For the FEEL‐KJ, the means of the primary strategies (aggregated from items) were used as indicators of adaptive and maladaptive strategies. Following the criteria of Brown [37], factor loadings lower than 0.30 were considered non‐salient and removed if necessary.

Combined measurement models incorporating all three instruments were then tested within a structural equation modelling (SEM) framework. Four models were specified, examining the interactions between attachment anxiety or attachment avoidance and adaptive or maladaptive emotion regulation strategies in relation to emotional eating. These measurement models served to define the latent variables and ensure that the indicators were valid before testing hypothesised structural and mediational relationships.

The main four structural models included a direct path from attachment to emotion regulation strategies and a mediated path from attachment to emotional eating through emotion regulation strategies. Then, following the same SEM approach and criteria, we explored how specific emotion regulation strategies (behavioural problem‐solving) and the diversity of emotion regulation strategies mediated the relations between attachment dimensions and emotional eating. Gender and age were included as covariates. The mediation effect was evaluated by constructing a 95% bias‐corrected bootstrap confidence interval (CI) (*k* = 10 000).

The goodness of fit of the models (CFA, measurement models and structural models) was interpreted based on the Chi‐square, the Comparative Fit Index (CFI), the Tucker‐Lewis Index (TLI), and the Root Mean Square Error of Approximation (RMSEA). CFI and TLI values close to 1.00 and RMSEA values lower than 0.08 were considered indicative of a good model fit [38].

Analyses were performed using the R‐package ‘lavaan’ [39]. Missing data were imputed using full information maximum likelihood (FIML) estimation. Outliers (±3 standard deviations) were modified with the weight modification method (winsorization). The syntax and code used for data analysis in this project are publicly available on the Open Science Framework (OSF) (https://osf.io/2skxt/?view_only=21a2454bee1143eda7f65b7c1309d60f).

## 3 Results

### 3.1 Sample Characteristics

The study included 772 participants aged 7–19 (295 boys, 477 girls; *M*_age_ = 14.01 years, SD = 2.43) enrolled in an inpatient Multidisciplinary Obesity Treatment program from 2013 to 2018. Most participants were adolescents (82.3%) and 17.7% were school‐age children (ages 7–12). Adjusted BMI (%BMI) data were available for 627 participants. In this subsample, the mean %BMI was 191.05 (SD = 30.38), with values ranging from 119.72 to 342.20, indicating severe obesity across the sample. Descriptive statistics and correlations among study variables can be found in the (Table S1).

### 3.2 Confirmatory Factor Analyses, Measurement Models and Structural Equation Models

For the ECR‐RC in the attachment anxiety subscale, items 17 and 21 were removed, while items 2, 6, 10, 12, 14, and 28 were removed from the attachment avoidance subscale (standardised factor loadings below 0.30). After rerunning the CFA, factor loadings were greater than 0.438, indicating a good relation between the items and the latent constructs. The fit was adequate, *χ* ^2^(349) = 1951.095, RMSEA = 0.080, SRMR = 0.068, CFI = 0.884, TLI = 0.874. The CFA for the DEBQ showed an adequate fit, *χ* ^2^(492) = 1991.047, RMSEA = 0.064, SRMR = 0.077, CFI = 0.894, TLI = 0.886. For emotional eating, the factor loadings were greater than 0.650. For the FEEL‐KJ, although the CFA yielded some suboptimal fit indices [*χ* ^2^(66) = 7049.990, RMSEA = 0.135, SRMR = 0.089, CFI = 0.892, TLI = 0.866], factor loadings exceeded 0.456, indicating strong latent variable relations and suggesting that the CFA model had a robust factor structure.

The fit indexes of the four measurement models and structural equation models were adequate (see Table 1). The factor loadings of observed variables on their latent factors were greater than 0.455. The significant effects of age and gender (see Table S2) justified their inclusion, but the main effects remained consistent after adjustment.

**TABLE 1:** Fit indices for the measurement and structural models.

		*χ* ^2^ (df)	RMSEA	SRMR	CFI	TLI
Measurement model	Att. anxiety–adaptive ERS	2022.311 (591)***	0.058	0.042	0.922	0.917
	Att. anxiety–maladaptive ERS	1731.677 (524)***	0.057	0.039	0.921	0.915
	Att. avoidance–adaptive ERS	1866.825 (461)***	0.065	0.038	0.926	0.920
	Att. avoidance–maladaptive ERS	1576.753 (402)***	0.064	0.039	0.926	0.920
Structural model	Att. anxiety–adaptive ERS	2174.641 (659)***	0.057	0.044	0.918	0.912
	Att. anxiety–maladaptive ERS	1868.185 (588)***	0.055	0.042	0.917	0.911
	Att. avoidance–adaptive ERS	2005.031 (521)***	0.063	0.039	0.922	0.916
	Att. avoidance–maladaptive ERS	1700.512 (458)***	0.061	0.039	0.922	0.916

Abbreviations: Att., attachment; EE, emotional eating; ERS, emotion regulation strategies.

*** *p* < 0.001.

### 3.3 Mediation Analyses by Attachment Dimension

#### 3.3.1 Attachment Anxiety

Attachment anxiety correlated positively with adaptive (*r*_latent_ = 0.162, *p* < 0.001), and maladaptive emotion regulation strategies (*r*_latent_ = 0.383, *p* < 0.001), and with emotional eating (*r*_latent_ = 0.284, *p* < 0.001). Adaptive emotion regulation strategies showed a weaker association with emotional eating (*r*_latent_ = 0.098, *p* = 0.012) than maladaptive strategies (*r*_latent_ = 0.348, *p* < 0.001).

**FIGURE 1:**
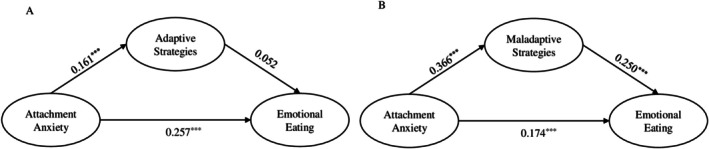
Structural model with adaptive emotion regulation strategies (A) and maladaptive emotion regulation strategies (B) as mediators of the impact of attachment anxiety on emotional eating. Standardised coefficients. Models controlled for age and gender. ^✝^ *p* < 0.1; *p < 0.05; **p < 0.01; ***p < 0.001.

Regarding adaptive strategies, in the mediation model, attachment anxiety predicted greater use of adaptive emotion regulation strategies (*β* = 0.161) (see Figure 1a). However, adaptive strategies did not significantly predict emotional eating (*β* = 0.052) (see Figure 1a). The indirect effect of attachment anxiety on emotional eating through adaptive strategies was not significant. The mediational model was examined with each of the specific emotion regulation strategies separately and the models showed an adequate fit (see Table S3). Among specific adaptive strategies, cognitive problem‐solving was a significant mediator (see Table S4). Attachment anxiety predicted greater use of this strategy, which in turn predicted increased emotional eating (6.383% of variance explained).

Attachment anxiety predicted greater use of maladaptive strategies (*β* = 0.366), which in turn significantly predicted emotional eating (*β* = 0.250) (see Figure 1b). Attachment anxiety also directly predicted emotional eating (*β* = 0.174 for the maladaptive model). Maladaptive emotion regulation strategies significantly mediated the effect of attachment anxiety on emotional eating, with the indirect effect accounting for 34.574% of the total effect (see Table 2). Specific mediating maladaptive strategies for attachment anxiety included giving up, withdrawal, self‐devaluation, and perseveration. Additionally, aggression was also a significant mediator in the case of attachment anxiety. The proportion of total effect explained by these indirect effects ranged from 12.234% to 25.532% (see Table S4).

**TABLE 2:** Structural model coefficients for the mediation models.

Model	Effect	*β*	SE	z	*p*	95% CI for indirect effect
Att. anxiety → adaptive ERS	Att. → EE	0.182	0.032	5.733	< 0.001	—
	Att. → ERS	0.096	0.024	4.079	< 0.001	—
	ERS → EE	0.062	0.047	1.323	0.186	—
	Total effect	0.188	0.032	5.936	< 0.001	—
	Indirect effect	0.006	0.005	1.214	0.225	[−0.003, 0.017]
Att. anxiety → maladaptive ERS	Att. → EE	0.123	0.032	3.808	< 0.001	—
	Att. → ERS	0.172	0.019	9.018	< 0.001	—
	ERS → EE	0.375	0.070	5.327	< 0.001	—
	Total effect	0.188	0.031	5.989	< 0.001	—
	Indirect effect	0.065	0.014	4.778	< 0.001	[0.040, 0.093]
Att. avoidance → adaptive ERS	Att. → EE	0.064	0.025	2.520	0.012	—
	Att. → ERS	0.160	0.023	7.106	< 0.001	—
	ERS → EE	0.071	0.051	1.403	0.161	—
	Total effect	0.075	0.024	3.174	0.002	—
	Indirect effect	0.011	0.009	1.332	0.183	[−0.004, 0.030]
Att. avoidance → maladaptive ERS	Att. → EE	0.041	0.024	1.733	0.083	—
	Att. → ERS	0.075	0.018	4.183	< 0.001	—
	ERS → EE	0.458	0.069	6.603	< 0.001	—
	Total effect	0.075	0.024	3.178	0.001	—
	Indirect effect	0.034	0.009	3.638	< 0.001	[0.017, 0.054]

*Note:* Unstandardised coefficients. Models controlled for age and gender.

Abbreviations: Att., attachment; EE, emotional eating; ERS, emotion regulation strategies.

#### 3.3.2 Attachment Avoidance

Attachment avoidance correlated positively with adaptive (*r*_latent_ = 0.311, *p* < 0.001) and maladaptive emotion regulation strategies (*r*_latent_ = 0.182, *p* < 0.001), and with emotional eating (*r*_latent_ = 0.116, *p* = 0.003). Adaptive emotion regulation strategies showed a weaker association with emotional eating (*r*_latent_ = 0.098, *p* = 0.013) than maladaptive strategies (*r*_latent_ = 0.348, *p* < 0.001).

**FIGURE 2:**
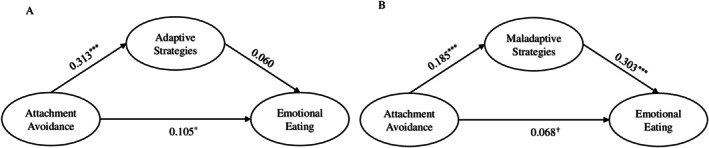
Structural model with adaptive emotion regulation strategies (A) and maladaptive emotion regulation strategies (B) as mediators of the impact of attachment avoidance on emotional eating. Standardised coefficients. Models controlled for age and gender. ^✝^ *p* < 0.1; *p < 0.05; **p < 0.01; ***p < 0.001.

In the mediation model, attachment avoidance predicted greater use of adaptive strategies (*β* = 0.313), but adaptive strategies did not significantly predict emotional eating (*β* = 0.060) (see Figure 2a). The indirect effect of attachment avoidance on emotional eating through adaptive strategies was not significant. As in the case of attachment anxiety, the mediational model was examined with each of the specific emotion regulation strategies separately and the models showed an adequate fit (see Table S3). Cognitive problem‐solving, as in the previous model, was a significant mediator and in the same direction as in the attachment anxiety model (32.000% of variance explained). Acceptance was a marginally significant mediator for attachment avoidance, suggesting that greater avoidance predicted greater use of acceptance, which weakly predicted emotional eating (21.333% of variance explained), in contrast to the attachment anxiety model in which it did not mediate.

Attachment avoidance also predicted a greater use of maladaptive strategies (*β* = 0.185), which significantly predicted emotional eating (*β* = 0.303) (see Figure 2b). As in the attachment anxiety model, adaptive strategies did not predict emotional eating, whereas maladaptive strategies did. However, in this case the direct effect of attachment avoidance on emotional eating in the maladaptive model (*β* = 0.068) did not reach statistical significance, remaining at a trend level (*p* = 0.083) and suggesting a full or near‐full mediation. Maladaptive emotion regulation strategies mediated the effect of attachment avoidance on emotional eating, with the indirect effect accounting for 45.333% of the total effect (see Table 2). Specific maladaptive strategies mediating the effect of attachment avoidance included giving up, withdrawal, self‐devaluation, and perseveration. The proportion of total effect explained by these indirect effects ranged from 26.667% to 46.667% (see Table S5). Unlike in the anxiety model, aggression was not a significant mediator.

### 3.4 Diversity of Emotion Regulation Strategies as Mediator

Finally, the mediational effects of the diversity index were explored, and it was found to be a significant mediator. For the attachment anxiety model, the fit indices indicated a good model fit: *χ* ^2^(459) = 1615.287, RMSEA = 0.059, SRMR = 0.042, CFI = 0.916, TLI = 0.910. The unstandardized coefficients for the three paths were positive and significant. Specifically, the direct effect of attachment anxiety on emotional eating was estimated to be 0.162 (*p* < 0.001), while the indirect effect through the diversity index was 0.026 (*p* = 0.002, 95% CI [0.011, 0.044]). The indirect path explained 13.830% of the total effect. For the attachment avoidance model, the fit was good too: *χ* ^2^(345) = 1460.749, RMSEA = 0.067, SRMR = 0.038, CFI = 0.923, TLI = 0.916. The coefficients for the paths were positive and significant except for the direct effect of attachment avoidance on emotional eating, which was not significant (*p* = 0.102), revealing total mediation. The indirect effect was 0.034 (*p* < 0.001, 95% CI [0.017, 0.054]) and explained 45.333% of the total effect.

## 4 Discussion

Previous studies highlighted the importance of understanding the underlying triggers of emotional eating [11]. The present study extends this work by examining whether maternal attachment insecurity (anxiety and avoidance) is associated with emotional eating in youths with obesity, and whether these associations are mediated by specific emotion regulation strategies. As emotion regulation strategies are an easier target for an intervention than attachment style itself, understanding their role may inform more effective clinical approaches.

Contrary to the original hypothesis that attachment insecurity would be negatively associated with adaptive emotion regulation and thus linked to less emotional eating, a positive association between attachment anxiety and adaptive strategies was observed. Attachment anxiety was associated with greater use of adaptive emotion regulation strategies, but these strategies were not associated with emotional eating. The only exception was the mediating role of cognitive problem‐solving. A possible interpretation is that youth high in attachment anxiety increase their efforts to manage distress cognitively because they doubt the availability or effectiveness of attachment support. In clinical samples of adolescents with underdeveloped emotion‐management skills, cognitive strategies may be applied ineffectively or inconsistently. In such cases, increased use of cognitive problem‐solving may not reduce negative affect and may even occur alongside emotional eating as a compensatory behaviour. This interpretation aligns with previous developmental evidence indicating that cognitive emotion‐regulation skills continue to mature during adolescence [40]. These unexpected results may also be interpreted considering the high emotional load typical of clinical samples of youths with obesity. Under such conditions, even strategies considered adaptive—such as cognitive problem‐solving—may be insufficient to regulate negative affect, which could explain their co‐occurring use with emotional eating.

Consistent with theoretical expectations, attachment anxiety was strongly linked to maladaptive emotion regulation strategies, which in turn partially mediated its association with emotional eating. The results align with findings on the mediating role of general emotion regulation difficulties in community samples of girls [27] and in adults with obesity [41]. The study by Vandewalle et al. [42], although focused on maternal rejection rather than attachment, also found that adaptive strategies did not mediate the relation with emotional eating in youths with obesity, whereas maladaptive strategies did. This pattern suggests that individuals with high attachment anxiety may be particularly vulnerable to using emotion regulation strategies that intensify rather than reduce distress—such as giving up, perseveration, self‐devaluation, or withdrawal. These strategies may ultimately increase the likelihood of eating in response to negative emotions. The presence of a significant direct effect alongside the indirect path implies that emotional eating may also serve as an immediate, compensatory behaviour to manage attachment‐related anxiety and affective arousal. Notably, the mediating role of aggression suggests that dysregulated externalising responses may coexist with internalising strategies, reflecting a broader pattern of emotional dysregulation among anxious youths. These findings align with previous evidence linking attachment anxiety to hyperactivation of the attachment system and heightened emotional reactivity [8], which together may sustain maladaptive coping cycles and reinforce emotional eating behaviours.

For attachment avoidance, there was again a positive association with adaptive strategies. Although these strategies are traditionally labelled as ‘adaptive’, their positive association with emotional eating may suggest that their effectiveness depends on the contextual or individual factors [23, 25]. The ‘adaptive/maladaptive’ distinction may not fully capture the complexity of emotion regulation processes, particularly in clinical samples of youths with obesity. In the mediation model, attachment avoidance predicted greater overall use of adaptive strategies, but adaptive strategies did not significantly predict emotional eating. The results on specific strategies parallel the pattern found for attachment anxiety, suggesting that while avoidant youths may employ adaptive strategies such as cognitive problem‐solving and acceptance, these strategies may not effectively reduce emotional eating. One possible explanation is that individuals high in avoidance tend to rely on self‐regulatory methods to maintain distance from emotional distress or interpersonal dependency. Such cognitive or acceptance‐based strategies may be used rigidly or defensively rather than flexibly, which may reduce their effectiveness.

Maladaptive emotion regulation strategies fully or almost fully accounted for the association between attachment avoidance and emotional eating. This suggests that emotional eating among avoidant individuals may result less from direct attachment‐related distress and more from the indirect effects of disengagement‐based coping mechanisms, such as giving up, withdrawal, perseveration, and self‐devaluation. Although avoidant youths tend to minimise emotional dependence and suppress distress, such suppression may paradoxically deplete regulatory resources and lead to rebound effects manifested in dysregulated behaviours like emotional eating. These findings align with previous research that shows an association of emotion regulation difficulties, such as emotional suppression, with emotional eating in youths [20]. The absence of a direct effect supports the idea that avoidant individuals' emotional eating emerges primarily through their habitual reliance on maladaptive regulatory responses. In contrast to the attachment anxiety model, aggression was not a significant mediator, likely reflecting avoidant individuals' preference for emotional disengagement rather than overt expression of distress.

The positive associations between attachment insecurities and emotion regulation strategies and their predictive role were further supported by the finding concerning the diversity of emotion regulation strategies. Due to a lack of confidence in the availability and support of their attachment figures, youths may resort to alternative strategies to manage their discomfort instead of seeking closeness [7, 16]. In the present study, greater diversity of emotion regulation strategies, in turn, predicted emotional eating. While a diverse and flexible repertoire of emotion regulation strategies is often considered adaptive [25], in this context, it may instead reflect a less efficient trial‐and‐error approach to emotion regulation. Consequently, such patterns of strategy use may contribute to emotional eating as a compensatory mechanism. This interpretation aligns with research linking emotion regulation diversity with depressive symptoms [24], suggesting that in clinical populations it may indicate emotional dysregulation rather than flexibility.

An important finding was the total mediation of the diversity index in the association between attachment avoidance and emotional eating. This suggests that the effect of attachment avoidance on emotional eating may operate primarily through the diversity of emotion regulation strategies. Avoidance, as a coping style, may offer short‐term relief but can lead to the accumulation of emotional tension when challenges persist. When emotions become overwhelming, individuals may turn to emotional eating as an automatic, learned response, particularly when their regulatory strategies are ineffective. Nevertheless, these results should be interpreted with caution. Although emotion regulation diversity accounted for a meaningful portion of the impact of attachment (especially avoidance) on emotional eating, it does not fully explain emotional eating. Other factors—such as individual differences, contextual stressors, or external influences—are likely to play additional roles. Future research should further explore these pathways and examine whether the role of emotion regulation diversity varies across developmental stages and clinical profiles.

### 4.1 Limitations and Future Directions

This study relied on self‐reports, which, while insightful, are prone to biases such as social desirability and inaccurate recall, particularly in youths [43]. Future research could incorporate multi‐informant reports or direct observations, such as mother–child interactions, performance in emotion regulation tasks, or eating behaviours. Additionally, the cross‐sectional design limits our ability to infer causality. Longitudinal studies with follow‐up assessments would help clarify the directionality and stability of these relations. Another limitation is the lack of a non‐clinical control group; including one in future studies could clarify patterns specific to clinical versus non‐clinical populations. In addition, adjusted BMI was not calculated for 19% of the participants due to anonymization of the dataset, which prevented data retrieval. Race/ethnicity and socioeconomic indicators were not available, as these variables were not included in the data collection protocol approved by the Ethics Committee. This limitation restricted the examination of potentially meaningful contextual influences that should be addressed in future studies. We focused on attachment to the mother, often considered as the most influential figure, but research on attachment to fathers and peers, who are also crucial for emotional development, is needed [44]. Future studies could also consider using categorical approaches to attachment, including disorganised forms, or explicitly measuring attachment security to obtain a more complete view of attachment. In terms of emotion regulation strategies, our study examined a wide range of strategies; however, there is still room to explore additional ones, such as self‐compassion. Finally, although only a small proportion of our sample consisted of school‐age children, it is important to note that emotion regulation processes evolve substantially from childhood to adolescence, and these developmental differences should be considered when interpreting our findings.

Despite these limitations, a key strength of our study is the inclusion of a large sample of youth with obesity, which enhances the reliability and relevance of our findings for similar populations and provides a strong foundation for the observed relations. Although some standardised coefficients were modest in size (*β* = 0.10–0.25), they fall within the range typically considered small to moderate [45, 46] and are consistent with previous mediation research involving complex psychosocial constructs such as attachment and emotion regulation. Additionally, although further research is needed on this matter, another contribution of the present study is the adoption of an innovative approach based on the diversity of emotion regulation strategies.

### 4.2 Conclusion

Overall, the present study helps to clarify the mechanisms underlying emotional eating in obesity, and provides support for comprehensive, multidimensional models that integrate emotion regulation and parental factors [47]. The results further underscore the relevance of attachment as an important factor to consider in the field of emotional eating. This finding is consistent with prior work in community samples [10], and highlights the role of early experiences and the family environment in the development of eating behaviours and obesity. Importantly, these findings could help design interventions and reduce the risk of obesity associated with emotional eating [26, 27]. Specifically, this study supports interventions that focus on attachment relationships and emotion regulation. Involving parents in therapy or improving family communication may strengthen familial support systems [48]. Additionally, interventions aimed at enhancing emotion regulation skills may benefit youths with obesity [47, 49], particularly by addressing maladaptive emotion regulation strategies. When appropriate, the use of well‐established problem‐solving techniques may help youths cope more effectively with painful emotional experiences. Assessing the youths' repertoire of emotion regulation strategies, and particularly the effectiveness of these strategies, may improve treatment outcomes. Interventions may also focus on fostering emotional and distress tolerance, particularly regarding physiological arousal, through techniques such as mindfulness and self‐compassion. Additionally, interventions may facilitate greater insight into the diversity of regulatory strategies that adolescents currently adopt, even when these strategies prove ineffective.

## Author Contributions

**Joana Gómez‐Odriozola:** conceptualization, methodology, formal analysis, funding acquisition, writing – original draft, writing – reviewing, and editing. **Jolien Braet:** conceptualization, methodology, formal analysis, funding acquisition, writing – reviewing, and editing. **Ine Verbiest:** conceptualization, provided statistical codes, funding acquisition, writing – reviewing, and editing. **Caroline Braet:** conceptualization, resources, supervision, writing – reviewing, and editing.

## Funding

This study was partially supported by the Basque Government (‘Culture, Cognition and Emotion’ Consolidated group: IT‐1797‐26), the Spanish Ministry of Science and Innovation (PID2023‐151085NB‐I00), the University of the Basque Country (UPV/EHU) (UES22/27), the Bijzonder Onderzoeksfonds UGent (BOF) (BOF20/DOC/015), and the Fonds Wetenschappelijk Onderzoek (FWO.3F0.2021.0087.01). This research was conducted within the framework of a research mobility stay funded by the University of the Basque Country (UPV/EHU), mobility grant MOV23/42.

## Disclosure

The hypotheses and data analysis plan are preregistered on the Open Science Framework under registration number: https://doi.org/10.17605/OSF.IO/FKQCM.

## Ethics Statement

The procedures of this study have been approved by the institutional research committee of the Ghent University Faculty of Psychology and Psychological Sciences (2015/88) and have been conducted in accordance with the ethical standards outlined in the 1964 Declaration of Helsinki and its subsequent amendments.

## Consent

Informed consent was obtained from all individual participants included in the study.

## Conflicts of Interest

The authors declare no conflicts of interest.

## Supporting information


**Table S1:** Descriptive statistics and correlations among study variables.
**Table S2:** Covariates in structural mediation models.
**Table S3:** Fit indices for the structural models for specific emotion regulation strategies.
**Table S4:** Coefficients for models of specific emotion regulation strategies mediating attachment anxiety and emotional eating.
**Table S5:** Coefficients for models of specific emotion regulation strategies mediating attachment avoidance and emotional eating.

## Data Availability

The data presented in this study are not available on request due to ethical consent restrictions; data syntaxes and analytic strategy are available in the Open Science Framework: https://osf.io/2skxt/?view_only=21a2454bee1143eda7f65b7c1309d60f.

## References

[1] L. R. Chawner and M. L. Filippetti , “A Developmental Model of Emotional Eating,” Developmental Review 72 (2024): 101133, 10.1016/j.dr.2024.101133.

[2] M. Frayn and B. Knäuper , “Emotional Eating and Weight in Adults: A Review,” Current Psychology 37 (2018): 924–933, 10.1007/s12144-017-9577-9.

[3] A. V. Valero‐García , M. Olmos‐Soria , J. Madrid‐Garrido , I. Martínez‐Hernández , and E. Haycraft , “The Role of Regulation and Emotional Eating Behaviour in the Early Development of Obesity,” International Journal of Environmental Research and Public Health 18, no. 22 (2021): 11884, 10.3390/ijerph182211884. 34831637 PMC8622852

[4] H. J. Webb , J. L. Kerin , and M. J. Zimmer‐Gembeck , “Increases in Emotional Eating During Early Adolescence and Associations With Appearance Teasing by Parents and Peers, Rejection, Victimization, Depression, and Social Anxiety,” Journal of Early Adolescence 41, no. 5 (2021): 754–777, 10.1177/0272431620950469.

[5] J. Bowlby , “Loss Sadness and Depression,” in Attachment and Loss, vol. III (Basic Books, 1980).

[6] M. Mikulincer and P. R. Shaver , “Adult Attachment and Emotion Regulation,” in Handbook of Attachment: Theory, Research, and Clinical Applications, ed. J. Cassidy and P. R. Shaver (Guilford Press, 2016), 507–533.

[7] A. Faber , L. Dube , and B. Knaeuper , “Attachment and Eating: A Meta‐Analytic Review of the Relevance of Attachment for Unhealthy and Healthy Eating Behaviors in the General Population,” Appetite 123 (2018): 410–438, 10.1016/j.appet.2017.10.043. 29183700

[8] T. Jewell , E. Apostolidou , K. Sadikovic , et al., “Attachment in Individuals With Eating Disorders Compared to Community Controls: A Systematic Review and Meta‐Analysis,” International Journal of Eating Disorders 56, no. 5 (2023): 888–908, 10.1002/eat.23922. 36916409

[9] F. Bahrami , R. Kelishadi , N. Jafari , Z. Kaveh , and O. Isanejad , “Association of Children's Obesity With the Quality of Parental‐Child Attachment and Psychological Variables,” Acta Paediatrica 102, no. 7 (2013): e321–e324, 10.1111/apa.12253. 23600901

[10] D. Maras , N. Obeid , M. Flament , et al., “Attachment Style and Obesity: Disordered Eating Behaviors as a Mediator in a Community Sample of Canadian Youth,” Journal of Developmental and Behavioral Pediatrics 37, no. 9 (2016): 762–770, 10.1097/DBP.0000000000000361. 27801724

[11] F. Favieri , A. Marini , and M. Casagrande , “Emotional Regulation and Overeating Behaviors in Children and Adolescents: A Systematic Review,” Behavioral Science 11, no. 1 (2021): 11, 10.3390/bs11010011. PMC7833366 33477932

[12] K. Brenning and C. Braet , “The Emotion Regulation Model of Attachment: An Emotion‐Specific Approach,” Personal Relationships 19, no. 3 (2012): 107–123, 10.1111/j.1475-6811.2012.01399.x.

[13] T. F. Heatherton and R. F. Baumeister , “Binge‐Eating as Escape From Self Awareness,” Psychological Bulletin 110, no. 1 (1991): 86–108, 10.1037/0033-2909.110.1.86. 1891520

[14] H. I. Kaplan and H. S. Kaplan , “The Psychosomatic Concept of Obesity,” Journal of Nervous and Mental Disease 125, no. 2 (1957): 181–201. 13481715 10.1097/00005053-195704000-00004

[15] A. Aldao , S. Nolen‐Hoeksema , and S. Schweizer , “Emotion‐Regulation Strategies Across Psychopathology: A Meta‐Analytic Review,” Clinical Psychology Review 30, no. 2 (2010): 217–237, 10.1016/j.cpr.2009.11.004. 20015584

[16] K. Van Durme , C. Braet , and L. Goossens , “Insecure Attachment and Eating Pathology in Early Adolescence: Role of Emotion Regulation,” Journal of Early Adolescence 35, no. 1 (2015): 54–78, 10.1177/0272431614523130.

[17] J. E. Cooke , L. B. Kochendorfer , K. L. Stuart‐Parrigon , A. J. Koehn , and K. A. Kerns , “Parent‐Child Attachment and Children's Experience and Regulation of Emotion: A Meta‐Analytic Review,” Emotion 19, no. 6 (2019): 1103, 10.1037/emo0000504. 30234329

[18] A. Iwanski , L. Lichtenstein , L. E. Mühling , and P. Zimmermann , “Effects of Father and Mother Attachment on Depressive Symptoms in Middle Childhood and Adolescence: The Mediating Role of Emotion Regulation,” Brain Sciences 11, no. 9 (2021): 1153, 10.3390/brainsci11091153. 34573173 PMC8469211

[19] A. W. Harrist , L. Hubbs‐Tait , G. L. Topham , L. H. Shriver , and M. C. Page , “Emotion Regulation Is Related to Children's Emotional and External Eating,” Journal of Developmental and Behavioral Pediatrics 34, no. 8 (2013): 557–565, 10.1097/DBP.0b013e3182a5095f. 24131878

[20] Q. Lu , F. Tao , F. Hou , Z. Zhang , and L. L. Ren , “Emotion Regulation, Emotional Eating and the Energy‐Rich Dietary Pattern: A Population‐Based Study in Chinese Adolescents,” Appetite 99 (2016): 149–156, 10.1016/j.appet.2016.01.011. 26792769

[21] L. H. Shriver , J. M. Dollar , S. D. Calkins , S. P. Keane , L. Shanahan , and L. Wideman , “Emotional Eating in Adolescence: Effects of Emotion Regulation, Weight Status and Negative Body Image,” Nutrients 13, no. 1 (2020): 79, 10.3390/nu13010079. 33383717 PMC7824438

[22] L. H. Shriver , J. M. Dollar , M. Lawless , et al., “Longitudinal Associations Between Emotion Regulation and Adiposity in Late Adolescence: Indirect Effects Through Eating Behaviors,” Nutrients 11, no. 3 (2019): 517, 10.3390/nu11030517. 30823405 PMC6470565

[23] G. A. Bonanno and C. L. Burton , “Regulatory Flexibility: An Individual Differences Perspective on Coping and Emotion Regulation,” Perspectives on Psychological Science 8, no. 6 (2013): 591–612, 10.1177/1745691613504116. 26173226

[24] A. Wen , L. Quigley , K. L. Yoon , and K. S. Dobson , “Emotion Regulation Diversity in Current and Remitted Depression,” Clinical Psychological Science 9, no. 4 (2021): 563–578, 10.1177/2167702620978616.

[25] A. Aldao , G. Sheppes , and J. J. Gross , “Emotion Regulation Flexibility,” Cognitive Therapy and Research 39, no. 3 (2015): 263–278, 10.1007/s10608-014-9662-4.

[26] R. Beijers , M. Miragall , Y. van den Berg , H. Konttinen , and T. van Strien , “Parent‐Infant Attachment Insecurity and Emotional Eating in Adolescence: Mediation Through Emotion Suppression and Alexithymia,” Nutrients 13, no. 5 (2021): 1662, 10.3390/nu13051662. 34068872 PMC8153636

[27] A. P. Schmitt , E. Hart , and C. M. Chow , “Attachment, Rumination, and Disordered Eating Among Adolescent Girls: The Moderating Role of Stress,” Eating and Weight Disorders 26 (2021): 1–9, 10.1007/s40519-020-01029-9. 33389702

[28] M. Van Winckel and E. Van Mil , “Wanneer Is Dik Te Dik?,” in Behandelingsstrategieën Bij Kinderen Met Overgewicht, ed. C. Braet and M. A. J. M. Van Winckel (Bohn Stafleu Van Loghum, 2001), 11–26.

[29] T. Naets , L. Vervoort , A. Tanghe , A. De Guchtenaere , and C. Braet , “Maladaptive Eating in Children and Adolescents With Obesity: Scrutinizing Differences in Inhibition,” Frontiers in Psychiatry 11 (2020): 309, 10.3389/fpsyt.2020.00309. 32425824 PMC7212434

[30] I. Verbiest , N. Michels , A. Tanghe , and C. Braet , “Inflammation in Obese Children and Adolescents: Association With Psychosocial Stress Variables and Effects of a Lifestyle Intervention,” Brain, Behavior, and Immunity 98 (2021): 40–47, 10.1016/j.bbi.2021.07.020. 34333112

[31] L. Vervoort , T. Naets , L. Goossens , et al., “Subtyping Youngsters With Obesity: A Theory‐Based Cluster Analysis,” Appetite 168 (2022): 105723, 10.1016/j.appet.2021.105723. 34606939

[32] M. S. Fritz and D. P. MacKinnon , “Required Sample Size to Detect the Mediated Effect,” Psychological Science 18, no. 3 (2007): 233–239, 10.1111/j.1467-9280.2007.01882.x. 17444920 PMC2843527

[33] C. Braet , L. Claus , L. Goossens , E. Moens , L. Van Vlierberghe , and B. Soetens , “Differences in Eating Style Between Overweight and Normal‐Weight Youngsters,” Journal of Health Psychology 13, no. 6 (2008): 733–743, 10.1177/1359105308093850. 18697886

[34] A. F. Hayes and J. J. Coutts , “Use Omega Rather Than Cronbach's Alpha for Estimating Reliability,” Communication Methods and Measures 14, no. 1 (2020): 1–24, 10.1080/19312458.2020.1718629.

[35] K. Brenning , B. Soenens , C. Braet , and G. Bosmans , “An Adaptation of the Experiences in Close Relationships Scale–Revised for Use With Children and Adolescents,” Journal of Social and Personal Relationships 28 (2011): 1048–1072, 10.1177/0265407511402418.

[36] C. Braet , “Het Meten Van Emotieregulatie Met de FEEL‐KJ: Een Nieuw Instrument,” Psychopraktijk 5, no. 5 (2013): 27–30.

[37] T. A. Brown , Confirmatory Factor Analysis for Applied Research (Guilford Press, 2015).

[38] L. T. Hu and P. M. Bentler , “Cutoff Criteria for Fit Indexes in Covariance Structure Analysis: Conventional Criteria Versus New Alternatives,” Structural Equation Modeling 6, no. 1 (1999): 1–55.

[39] Y. Rosseel , “Lavaan: An R Package for Structural Equation Modeling,” Journal of Statistical Software 48, no. 2 (2012): 1–36, 10.18637/jss.v048.i02.

[40] E. A. Skinner and M. J. Zimmer‐Gembeck , “Age Differences and Changes in Ways of Coping Across Childhood and Adolescence,” in The Development of Coping: Stress, Neurophysiology, Social Relationships, and Resilience During Childhood and Adolescence, ed. E. A. Skinner and M. J. Zimmer‐Gembeck (Springer, 2016), 53–62.

[41] M. Taube‐Schiff , J. Van Exan , R. Tanaka , S. Wnuk , R. Hawa , and S. Sockalingam , “Attachment Style and Emotional Eating in Bariatric Surgery Candidates: The Mediating Role of Difficulties in Emotion Regulation,” Eating Behaviors 18 (2015): 36–40, 10.1016/j.eatbeh.2015.03.011. 25875114

[42] J. Vandewalle , E. Moens , and C. Braet , “Comprehending Emotional Eating in Obese Youngsters: The Role of Parental Rejection and Emotion Regulation,” International Journal of Obesity 38, no. 4 (2014): 525–530, 10.1038/ijo.2013.233. 24418843

[43] L. J. Crockett , J. E. Schulenberg , and A. C. Petersen , “Congruence Between Objective and Self‐Report Data in a Sample of Young Adolescents,” Journal of Adolescent Research 2, no. 4 (1987): 383–392, 10.1177/074355488724006.

[44] N. Islamiah , S. Breinholst , M. A. Walczak , and B. H. Esbjørn , “The Role of Fathers in Children's Emotion Regulation Development: A Systematic Review,” Infant and Child Development 32, no. 2 (2023): e2397, 10.1002/icd.2397.

[45] J. Cohen , Statistical Power Analysis for the Behavioral Sciences, 2nd ed. (Lawrence Erlbaum Associates, 1988).

[46] R. B. Kline , Principles and Practice of Structural Equation Modeling, 4th ed. (Guilford Press, 2015).

[47] E. Aparicio , J. Canals , V. Arija , S. De Henauw , and N. Michels , “The Role of Emotion Regulation in Childhood Obesity: Implications for Prevention and Treatment,” Nutrition Research Reviews 29, no. 1 (2016): 17–29, 10.1017/S0954422415000153. 27045966

[48] J. A. Skelton , C. Buehler , M. B. Irby , and J. G. Grzywacz , “Where Are Family Theories in Family‐Based Obesity Treatment? Conceptualizing the Study of Families in Pediatric Weight Management,” International Journal of Obesity 36, no. 7 (2012): 891–900. 22531090 10.1038/ijo.2012.56 PMC3977510

[49] T. Debeuf , S. Verbeken , B. Volkaert , et al., “Emotion Regulation Training as an Add‐On in the Treatment of Obesity in Young Adolescents: A Randomized Controlled Superiority Trial,” Behavior Therapy 55 (2024): 839–855, 10.1016/j.beth.2023.12.005. 38937054

