# Supplementary material for: Unravelling the Ties: How Attachment Styles and Emotion Regulation Fuel Emotional Eating in Youth With Obesity—A Clinical Sample Study
Source: Pediatr Obes. 2026 Feb 9;21(2):e70095. doi: 10.1111/ijpo.70095 (PMC12887149; doi:10.1111/ijpo.70095)
Supplement: Supplementary file 1 — Table S1: Descriptive statistics and correlations among study variables. Table S2: Covariates in structural mediation models. Table S3: Fit indices for the structural models for specific emotion regulation strategies. Table S4: Coefficients for models of specific emotion regulation strategies mediating attachment anxiety and emotional eating. Table S5: Coefficients for models of specific emotion regulation strategies mediating attachment avoidance and emotional eating. [file IJPO-21-e70095-s001.pdf]

Supplementary Material

Unraveling the Ties: How Attachment Styles and Emotion Regulation Fuel Emotional Eating in Youth with Obesity– A Clinical Sample Study

Joana Gómez-Odriozola[1], Jolien Braet[2], Ine Verbiest[2], & Caroline Braet[2]

[1]Department of Clinical and Health Psychology & Research Methods, University of the Basque Country UPV/EHU, Spain

[2]Department of Developmental, Personality, and Social Psychology, Ghent University, Belgium

Correspondence concerning this article should be addressed to Joana Gómez-Odriozola, Department of Clinical and Health Psychology & Research Methods, University of the Basque Country UPV/EHU, Spain, e-mail: joanamaialen.gomez@ehu.eus

Table S1

Descriptive Statistics and Correlations among Study Variables

		1	2	3	4	5	6	7	8	9	10
1	Emotional Eating	--									
2	Attachment Anxiety	.294***	--								
3	Attachment Avoidance	.186***	.507***	--							
4	Adaptive Strategies	.101**	.217***	.317***	--						
5	Maladaptive Strategies	.304***	.382***	.270***	.445***	--					
6	Problem-oriented action	.086*	.196***	.285***	.924***	.396***	--				
7	Distraction	.045	.161***	.276***	.878***	.281***	.787***	--			
8	Good Humor	.040	.137***	.272***	.882***	.292***	.806***	.818***	--		
9	Acceptance	.124***	.229***	.288***	.881***	.379***	.820***	.751***	.727***	--	
10	Forget	.070	.209***	.289***	.869***	.463***	.764***	.674***	.697***	.721***	--
11	Cognitive problem-solving	.155***	.208***	.290***	.830***	.482***	.764***	.632***	.667***	.675***	.681***
12	Reevaluation	.100**	.186***	.212***	.787***	.429***	.650***	.622***	.570***	.653***	.731***
13	Giving up	.212***	.325***	.228***	.213***	.825***	.182***	.099**	.092	.194***	.264***
14	Aggression	.174***	.202***	.117**	.318***	.614***	.289***	.262***	.279***	.268***	.284***
15	Withdrawal	.273***	.299***	.217***	.231***	.815***	.192***	.106**	.098**	.201***	.296***
16	Self-devaluation	.253***	.343***	.217***	.434***	.790***	.382***	.266***	.263***	.380***	.433***
17	Perseveration	.262***	.304***	.265***	.517***	.828***	.474***	.337***	.379***	.409***	.504***
18	Social support	.071	.153***	.260***	.605***	.338***	.566***	.542***	.590***	.497***	.489***
19	Expression	.193***	.256***	.278***	.375***	.583***	.338***	.293***	.331***	.312***	.371***
20	Control over emotion	.104**	.261***	.251***	.424***	.604***	.376***	.314***	.251***	.385***	.453***
21	Age	.166***	.074*	-.014	.036	.090*	.036	-.008	-.033	.024	.028
22	Gender	.134***	.076*	.055	.030	.192***	.015	-.024	.020	.019	.080*
	Mean	2.468	2.368	3.411	2.795	2.518	2.824	2.923	2.938	2.763	2.850
	SD	1.049	1.206	1.438	0.846	0.725	0.945	1.038	1.107	0.916	0.946

$^{*}p < .05$; $^{**}p < .01$; $^{***}p < .001$

Table S1 (continued)

Descriptive Statistics and Correlations among Study Variables

	11	12	13	14	15	16	17	18	19	20	21
11 Cognitive problem-solving	--										
12 Reevaluation	.593***	--									
13 Giving up	.223***	.250***	--								
14 Aggression	.275***	.278***	.418***	--							
15 Withdrawal	.255***	.266***	.650***	.327***	--						
16 Self-devaluation	.482***	.434***	.538***	.251***	.607***	--					
17 Perseveration	.610***	.419***	.605***	.381***	.570***	.641***	--				
18 Social support	.560***	.395***	.180***	.469***	.071	.171***	.407***	--			
19 Expression	.352***	.282***	.487***	.646***	.355***	.273***	.495***	.585***	--		
20 Control over emotion	.384***	.413***	.478***	.148***	.592***	.586***	.527***	.042	.106**	--	
21 Age	.110**	.078*	.013	-.045	.125***	.167***	.083*	-.105**	-.139***	.105**	--
22 Gender	.061	.001	.167***	.076*	.156***	.140***	.199***	.101**	.146***	.103**	.040
Mean	2.803	2.459	2.476	2.232	2.511	2.638	2.738	2.616	2.507	2.891	14.010
SD	1.003	0.886	0.891	0.930	0.940	0.967	0.951	1.069	0.962	0.935	2.430

$^{*}p < .05$; $^{**}p < .01$; $^{***}p < .001$

Note. To assist interpretation, we provide here references to published studies and the instrument manuals that report descriptive values for child and adolescent samples. These sources can be used as contextual benchmarks; however, they do not constitute universal norms applicable without adjustment. In particular, manuals and published descriptive studies for the FEEL-KJ include age- and gender-dependent reference values for Dutch/Flemish populations, and validation studies report typical ranges and psychometric properties for community and clinical samples. Because available reference values are age- and gender-dependent and different studies sometimes use different scoring conventions (e.g., summed vs. mean scores), direct group-level comparisons with published samples are not straightforward and should be interpreted with caution. For these reasons we report our data in the original metric and invite readers to consult the cited references for more detailed, age- and gender-specific benchmarks.

Brenning, K, Soenens, B, Braet, C, Bosmans, G. An adaptation of the experiences in close relationships scale–Revised for use with children and adolescents. *J Soc Pers Relat.* 2011;28:1048-1072. doi:10.1177/0265407511402418.

Braet C, Claus L, Goossens L, Moens E, Van Vlierberghe L, Soetens B. Differences in eating style between overweight and normal-weight youngsters. J *Health Psychol.* 2008;13(6):733-743. doi:10.1177/1359105308093850

Braet C, Van Beveren ML, Cracco E, Theuwis L, Grob A, Smolenski C. *FEEL-KJ: vragenlijst over emotieregulatie bij kinderen en jongeren. Amsterdam:* Hogrefe; 2020.

Table S2

Covariates in Structural Mediation Models

	Age		Gender	
	Impact on EE	Impact on ERS	Impact on EE	Impact on ERS
Att. anxiety and Adaptive ERS	0.064^{***}	0.000	0.271^{***}	0.030
Att. anxiety and Maladaptive ERS	0.055^{**}	0.025^{*}	0.171^{*}	0.271^{***}
Att. avoidance and Adaptive ERS	0.076^{***}	0.013	0.292^{***}	0.028
Att. avoidance and Maladaptive ERS	0.061^{**}	0.037^{**}	0.162^{*}	0.290^{***}

Note. Unstandardized coefficients. Gender coded as 1 = Boy and 2 = Girl. Att. = attachment, ERS = emotion regulation strategies, EE = emotional eating

$^{*}p < .05$; $^{**}p < .01$; $^{***}p < .001$

Table S3

Fit Indices for the Structural Models for Specific Emotion Regulation Strategies

Model	χ^2 (df)		RMSEA	SRMR	CFI	TLI
Attachment anxiety						
Problem-oriented action	1837.340(623)	***	0.052	0.040	0.920	0.915
Distraction	1941.100(623)	***	0.054	0.042	0.916	0.910
Good Humor	2053.518(623)	***	0.056	0.044	0.912	0.906
Acceptance	1914.844(623)	***	0.054	0.041	0.914	0.909
Forget	1925.849(623)	***	0.054	0.041	0.914	0.909
Cognitive problem-solving	1921.334(623)	***	0.054	0.044	0.917	0.911
Reevaluation	1873.413(623)	***	0.053	0.041	0.918	0.912
Giving up	1851.272(623)	***	0.052	0.040	0.917	0.911
Aggression	2128.450(623)	***	0.058	0.042	0.902	0.895
Withdrawal	1931.517(623)	***	0.054	0.042	0.913	0.907
Self-devaluation	1983.530(623)	***	0.055	0.042	0.912	0.906
Perseveration	1996.243(623)	***	0.055	0.043	0.909	0.903
Social support	1868.565(623)	***	0.053	0.041	0.922	0.916
Expression	2136.652(623)	***	0.058	0.047	0.902	0.895
Control over emotion	1851.783(623)	***	0.052	0.041	0.916	0.911
Attachment avoidance						
Problem-oriented action	1665.187(489)	***	0.058	0.037	0.926	0.920
Distraction	1736.629(489)	***	0.060	0.038	0.923	0.917
Good Humor	1835.563(489)	***	0.062	0.038	0.920	0.914
Acceptance	1745.247(489)	***	0.060	0.039	0.920	0.914
Forget	1748.244(489)	***	0.060	0.036	0.920	0.914
Cognitive problem-solving	1733.274(489)	***	0.059	0.039	0.923	0.917
Reevaluation	1708.454(489)	***	0.059	0.037	0.923	0.917
Giving up	1742.201(489)	***	0.060	0.041	0.918	0.912
Aggression	1965.469(489)	***	0.065	0.041	0.907	0.900
Withdrawal	1770.197(489)	***	0.060	0.041	0.918	0.912
Self-devaluation	1810.460(489)	***	0.061	0.039	0.917	0.911
Perseveration	1843.633(489)	***	0.062	0.041	0.914	0.907
Social support	1716.629(489)	***	0.059	0.037	0.926	0.920
Expression	1966.360(489)	***	0.063	0.043	0.908	0.900
Control over emotion	1738.144(489)	***	0.060	0.039	0.919	0.912

$^{*}p < .05$; $^{**}p < .01$; $^{***}p < .001$

Table S4

Coefficients for Models of Specific Emotion Regulation Strategies Mediating Attachment Anxiety and Emotional Eating

Model	Att. anxiety-EE	Att. anxiety-ERS	ERS- EE	Total effect	Indirect effect	95% CI for indirect effect	
Problem-oriented action	0.183***	0.063**	0.081	0.188***	0.005	-0.003	0.016
Distraction	0.186***	0.079**	0.021	0.188***	0.002	-0.006	0.010
Good Humor	0.186***	0.056*	0.029	0.188***	0.002	-0.003	0.008
Acceptance	0.178***	0.121***	0.085†	0.188***	0.010	-0.001	0.024
Forget	0.185***	0.085***	0.031	0.188***	0.003	-0.008	0.014
Cognitive problem-solving	0.176***	0.093***	0.130**	0.188***	0.012*	0.002	0.025
Reevaluation	0.182***	0.090***	0.068	0.188***	0.006	-0.004	0.018
Giving up	0.147***	0.177***	0.230***	0.188***	0.041**	0.018	0.067
Aggression	0.165***	0.122***	0.185**	0.188***	0.023**	0.008	0.041
Withdrawal	0.140***	0.147***	0.325***	0.188***	0.048***	0.027	0.072
Self-devaluation	0.144***	0.196***	0.224***	0.188***	0.044***	0.022	0.070
Perseveration	0.143***	0.125***	0.357***	0.188***	0.045***	0.025	0.068
Social support	0.184***	0.073**	0.057	0.188***	0.004	-0.003	0.014
Expression	0.162***	0.096***	0.265**	0.188***	0.025**	0.010	0.045
Control over emotion	0.182***	0.154***	0.034	0.188***	0.005	-0.010	0.021

Note. Unstandardized coefficients. Models controlled for age and gender. Att. = attachment, ERS = emotion regulation strategies, EE = emotional eating

†$p < .1$ *$p < .05$; **$p < .01$; ***$p < .001$

Table S5

Coefficients for Models of Specific Emotion Regulation Strategies Mediating Attachment Avoidance and Emotional Eating

Model	Att. avoidance-EE	Att. avoidance-ERS	ERS- EE	Total effect	Indirect effect	95% CI for indirect effect	
Problem-oriented action	0.065**	0.110***	0.090	0.075**	0.010	-0.006	0.028
Distraction	0.073**	0.164***	0.011	0.075**	0.003	-0.013	0.019
Good Humor	0.073**	0.163***	0.014	0.075**	0.002	-0.013	0.019
Acceptance	0.059*	0.150***	0.110*	0.075**	0.016†	0.001	0.035
Forget	0.070**	0.122***	0.048	0.075**	0.006	-0.009	0.023
Cognitive problem-solving	0.052*	0.166***	0.143**	0.075**	0.024*	0.006	0.045
Reevaluation	0.066**	0.098***	0.093	0.075**	0.009	-0.002	0.023
Giving up	0.056*	0.064**	0.312***	0.075**	0.020**	0.007	0.036
Aggression	0.069**	0.027	0.246***	0.075**	0.007	-0.004	0.018
Withdrawal	0.049*	0.068***	0.391***	0.075**	0.027**	0.011	0.044
Self-devaluation	0.053*	0.077**	0.239***	0.075**	0.023**	0.009	0.039
Perseveration	0.041†	0.082***	0.422***	0.075**	0.035***	0.018	0.056
Social support	0.067**	0.155***	0.055	0.075**	0.009	-0.007	0.026
Expression	0.045†	0.094***	0.321***	0.075**	0.030**	0.015	0.049
Control over emotion	0.068**	0.101***	0.078	0.075**	0.008	-0.002	0.020

Note. Unstandardized coefficients. Models controlled for age and gender. Att. = attachment, ERS = emotion regulation strategies, EE = emotional eating

†$p < .1$ *$p < .05$; **$p < .01$; ***$p < .001$
